# Exploring Disparities in Gill Physiological Responses to NaHCO_3_-Induced Habitat Stress in Triploid and Diploid Crucian Carp (*Carassius auratus*): A Comprehensive Investigation Through Multi-Omics and Biochemical Analyses

**DOI:** 10.3390/metabo15010005

**Published:** 2024-12-30

**Authors:** Shicheng Han, Lin Han, Fangying Yuan, Wenzhi Liu, Jing Wang, Xiaofeng Jin, Yanchun Sun

**Affiliations:** 1 Laboratory of Quality & Safety Risk Assessment for Aquatic Products (Harbin), Heilongjiang River Fisheries Research Institute of Chinese Academy of Fishery Sciences, Ministry of Agriculture and Rural Areas, Harbin 150070, China; hljhsc@126.com (S.H.); hanlin200112@163.com (L.H.); yuanfy8013@163.com (F.Y.); liuwenzhi0457@163.com (W.L.); jinxf_2023@163.com (X.J.); 2 Department of Food Science and Engineering, College of Food Science and Technology, Shanghai Ocean University, Shanghai 201306, China; 3 Department of Chemical Engineering and Technology, College of Materials and Chemical Engineering, Harbin University of Science and Technology, Harbin 150080, China; wangj199808@163.com

**Keywords:** crucian carp (*Carassius auratus*), diploid, triploid, carbonate alkalinity, metabolomics, transcriptomics

## Abstract

**Background**: Owing to the progressive rise in saline waters globally, resulting in detrimental impacts on freshwater aquaculture, the underlying molecular distinctions governing the response to alkaline stress between diploid and triploid crucian carp remain unknown. **Methods**: This investigation explores the effects of 20 and 60 mmol NaHCO_3_ stress over 30 days on the gills of diploid and triploid crucian carp, employing histological, biochemical, and multi-omic analyses. **Results**: Findings reveal structural damage to gill lamellas in the examined tissue. Diploid crucian carp exhibit heightened activities of superoxide dismutase (SOD), catalase (CAT), glutathione peroxidase (GSH-Px), and alkaline phosphatase (AKP), alongside lower malondialdehyde (MDA) and urea nitrogen (BUN) levels compared to triploid counterparts. Metabolomic investigations suggest alterations in purine metabolism, lipid metabolism, sphingolipid metabolism, and aminoglycan and nucleotide sugar metabolism following NaHCO_3_ exposure. Transcriptomic data indicate differential expression of genes associated with nitrogen metabolism, complement and coagulation cascades, IL-17 signaling pathways, and Toll-like receptor signaling pathways. **Conclusions**: Overall, NaHCO_3_-induced stress leads to significant gill tissue damage, accompanied by reactive oxygen species (ROS) production causing oxidative stress and disruptions in lipid metabolism in crucian carp. Furthermore, an inflammatory response in gill cells triggers an immune response. Diploid crucian carp exhibit superior antioxidant and immune capacities compared to triploid counterparts, while also displaying reduced inflammatory responses in vivo. Notably, diploid carp efficiently excrete excess BUN through purine metabolism, mitigating protein metabolism and amino acid imbalances caused by BUN accumulation. This enables them to allocate less energy for coping with external environmental stress, redirecting surplus energy toward growth and development. The above results indicate that diploid organisms can better adapt to saline–alkaline environments. Overall, this study provides novel perspectives into species selection of crucian carp of different ploidy in saline–alkaline waters.

## 1. Introduction

Saline–alkaline waters account for a large proportion of the world’s water resources, with high alkalinity being one of its main characteristics [1]. In China alone, there are approximately 46 million hectares of low-lying saline alkali land, primarily marked by substantial carbonate alkalinity (CA) [2]. Among the various stressors faced by aquatic organisms, high CA stands out as a common challenge [3]. Typically characterized by heightened pH levels, bicarbonate (HCO_3_^−^), and carbonate (CO_3_^2−^) concentrations, alkaline water inhibits ammonia excretion and promotes increased CO_2_ excretion in fish living in such environments [4]. These alterations can profoundly impact the growth, metabolism, and development of aquatic organisms, leading to diminished biomass and significant shifts in species composition [5]. Moreover, recent investigations highlight the expanding salinization of freshwater as a pressing environmental concern, posing a substantial threat to global freshwater ecosystems [6].

The impact of carbonate alkaline water on the physiological functions of aquatic organisms is profound [7]. Previous research has demonstrated that alkaline stress induces changes in vivo accumulation of reactive oxygen species (ROS), oxidative damage, and histopathological alterations in Amur minnow (*Phoxinus lagowskii*) [1]. Additionally, after 30 days of saline stress, crucian carp kidneys exhibited varying degrees of damage, characterized by glomerular atrophy and swelling, renal tubular degranulation, obstruction and degeneration, renal interstitial edema, renal cell proliferation, and necrosis [8]. Similarly, elevated carbonate alkalinity in Cyprinus carpio Songpu resulted in a significant decrease in weight gain, a notable increase in feed conversion, altered composition of the intestinal microbiota, and a reduction in the number of probiotic bacteria. Consequently, this led to a diminished growth rate, accompanied by a decrease in the antioxidant capacity of Songpu mirror carp and subsequent oxidative damage [9]. Despite these findings, the majority of studies have focused on physiological and biochemical indicators, as well as functional analyses of proteins and gut microbiota. To date, the comprehensive molecular regulatory mechanisms governing genetic and metabolic adaptation to alkaline water in aquatic organisms remain incompletely clear.

Crucian carp, a member of the Carassidae family, is extensively studied within the scientific community [10,11,12,13]. The triploid crucian carp is a hybrid of female crucian carp with male allotetraploids [12,14]. Previous investigations have explored the distinct responses to environmental stress among fish with different ploidy levels. For instance, ploidy turbot (*Scophthalmus maximus*) groups reared at 10 °C exhibited the lowest growth in length and weight, with the triploid group demonstrating a significantly lower growth rate in weight compared to the diploid group [15]. Under fishing pressure, hepatic glucose metabolism pathways differed between triploid and diploid rainbow trout (*Oncorhynchus mykiss*), with the triploids displaying higher basic glucose metabolism intensity than diploids. Additionally, fishing stress induced an earlier state of immunosuppression in triploid trout compared to diploid trout [16]. In the case of triploid and diploid brook charr (*Salvelinus fontinalis*), increasing the temperature from 15 °C to 18 °C improved the hypoxia tolerance of triploids in high-temperature-induced anoxic environments. However, triploids exhibited lower hypoxia tolerance than diploids at the same temperatures [17]. Notably, while progress has been made in studying different ploidy fishes under various environmental stresses, investigations specifically addressing the responses of diploid and triploid crucian carp to alkaline carbonate stress are lacking in the literature.

In recent years, the rapid development of various omics techniques has made it possible to comprehensively study the responses of aquatic organisms to external environmental stresses [18,19]. Previous studies leveraged transcriptomics to unravel the response mechanisms in hybrid tilapia (*Oreochromis mossambicus* female × *O. urolepis hornorum* male) [20], Nile tilapia (*Oreochromis niloticus*) [21], mussel (*Solenaia oleivora*) [22], and other aquatic organisms exposed to saline–alkaline stress. While transcriptome sequencing provides insights into gene expression, it is not sufficient to identify changes in organismal metabolite levels [23]. Metabolomics, a technological tool developed after genomics and proteomics, sensitively captures the impact of environmental factors by tracking alterations in endogenous metabolites [24]. Existing literature reports researchers exploring the response mechanisms of aquatic organisms to carbonate alkaline stress through metabolomics [25]. However, single-omics techniques have inherent limitations that are barriers to a comprehensive analysis of the molecular mechanisms underlying environmental stress [26]. Consequently, there is a growing recognition of the necessity for an integrated approach that combines metabolomics and transcriptomics to enable a more systematic analysis of gene and metabolite responses to alkaline stress in triploid and diploid crucian carp.

Among the functional organs of fish, gills play pivotal roles in respiration, ionic and osmotic regulation, ion balance, and excretion of waste nitrogen, establishing direct and continuous interactions with the surrounding environment [27]. The abundance of capillaries in the gills facilitates direct exchange with the water column, rendering them highly responsive to variations in water quality [28]. Consequently, gills are frequently regarded as the primary focus in studies on culturing under alkaline water conditions [1,29,30,31]. In this study, we employed a multi-omics approach, integrating metabolomics and transcriptomics, to scrutinize the distinctions in potential molecular mechanisms exhibited by triploid and diploid crucian carp in response to carbonate alkaline stress. This study provides a preliminary theoretical basis for species selection of different ploidy of crucian carp for aquaculture in saline–alkaline waters.

## 2. Materials and Methods

### 2.1. Chemicals and Reagents

NaHCO_3_ was obtained from Shanghai Aladdin Company (Shanghai, China). The reagent kits used for biochemical assays, including superoxide dismutase (SOD), catalase (CAT), glutathione peroxidase (GSH-Px), malondialdehyde (MDA), alkaline phosphatase (AKP), and urea nitrogen (BUN), were purchased from Nanjing Jiancheng Bioengineering Institute (Nanjing, China).

The equipment used for biochemical analysis is as follows: Electronic Analytical Balance (XS205DY, Mettler Toledo, Switzerland, sensitivity 0.01 mg), High-throughput tissue grinder (SCIENTZ-48L, SCIENTZ, Ningbo, China, sensitivity 5 μm), Microplate reader (SpectraMax Plus 384, Molecular Devices, San Jose, CA, USA, sensitivity 0.0001), Centrifuge (Allegra X-30R, Beckman, Pasadena, CA, USA, maximum rotation speed 18,000 rpm).

### 2.2. Experimental Animals and Design

Diploid male fish and triploid crucian carp (2 years old) with a mean weight of 140.1 ± 6.2 g and length of 17.83 ± 1.28 cm were sourced from Hulan Experimental Station, Heilongjiang Fisheries Research Institute, Chinese Academy of Fisheries Science. Ploidy determination via flow cytometry classified the fish into diploid crucian carp (with a cell chromosome count of 2n, specifically 100, abbreviated as 2n) and triploid crucian carp (with a cell chromosome count of 3n, specifically 150, abbreviated as 3n). All animal procedures in this study adhered to the Guidelines for Care and Use of Laboratory Animals of Heilongjiang River Fisheries Research Institute of the Chinese Academy of Fishery Sciences and received approval from the Animal Ethics Committee of Heilongjiang River Fisheries Research Institute of the Chinese Academy of Fishery Sciences (No. 9972).

Following a two-week acclimatization period, 270 fish were randomly distributed among 18 fiberglass tanks (each with a capacity of 200 L and dimensions of 100 × 50 × 40 cm). The allocation maintained an equal division between triploid and diploid crucian carp, with 15 fish in each tank. The tanks were organized into distinct groups: a control group with fresh dechlorinated water (Con 2n, Con 3n), a base concentration group with 20 mmol/L NaHCO_3_ (CA20 2n, CA20 3n), and another base concentration group with 60 mmol/L NaHCO_3_ (CA60 2n, CA60 3n). The experimental design is shown in Table 1. The chosen CA concentrations were informed by prior experiments conducted on data from highly alkaline waters in Chinese aquatic ecosystems [32]. Throughout the domestication and experimental phases, water temperature was maintained at 23.0 ± 1.0 °C through the use of an automatic temperature controller, with a daily light cycle of 12 h. Crucians were fed twice daily at 8:00 a.m. and 5:00 p.m. with a commercial pellet feed at 3% of the total fish weight (Tongwei Feed Company, Tianjin, China). Daily, one-third of the tank water was replaced, and the corresponding amount of CA was added and monitored to uphold the designated concentration.

On the 30th day of the experiment, eight fish were randomly chosen from each tank, totaling 144 fish. Subsequently, all fish were euthanized using MS-222 anesthesia (100 mg/L, Sigma, St. Louis, MO, USA). Blood samples were drawn from the tail vein and placed into centrifuge tubes. Gill tissue, weighing a total of 24 gills per group, was dissected on ice. One of the gills was embedded in paraffin for subsequent histological analysis, while the remaining gills were promptly frozen in liquid nitrogen and stored at −80 °C for future use. Fifteen gills were allocated for UPLC-Q-TOF/MS metabolomic analysis, five for biochemical analysis, and the remaining three for transcriptomic analysis.

### 2.3. Tissue Sectioning and Photographic Observation

Following the fixation of gill tissue in 4% paraformaldehyde (volume fraction) for 24 h, a gradual dehydration process was performed using ethanol at varying concentrations (50%, 70%, 80%, 95%, and 100%). The tissues were then cleared in xylene and embedded in paraffin. Subsequently, sections with a thickness of 5 µm were obtained using a sectioning machine (RM2235, LEICA, Wetzlar, Germany) and stained with hematoxylin and eosin (H&E). Finally, the stained gill tissue sections were captured under a light microscope (BX53, OLYMPUS, Tokyo, Japan) at 400× magnification, and images were recorded using a DP73 camera (OLYMPUS, Tokyo, Japan) [33].

### 2.4. Biochemical Indicators Detection

Blood was stored at 4 °C for 3 h protected from light, followed by centrifugation at 2000× *g* for 10 min to obtain serum. Serum was used to analyze urea nitrogen (BUN) levels. Gill tissue samples and physiological saline were mixed in a 1:9, *w*/*v* ratio. The supernatant was collected by centrifugation at 2500× *g* for 10 min at 4 °C to further determine the activity of SOD, CAT, GSH-Px, AKP, and MDA levels.

### 2.5. Metabolomics Analysis

Frozen gill samples were thawed at 4 °C. Then, 60 mg samples were placed in a 1.5 mL microcentrifuge tube with three tiny steel beads and 1000 µL of extraction solution (methanol/water = 4:1). The samples were homogenized at 60 Hz for 3 min and sonicated in an ice bath for 20 min. Post-homogenization, the extract was centrifuged at 4 °C at 13,000 rpm for 15 min. Subsequently, the supernatant (800 microliters) was collected, filtered through 0.22 μm micrometer filters, and transferred to automatic injection vials for metabolic analysis. During the preparation of quality control (QC) samples, all individual samples were mixed in equal volumes, following the pre-processing method outlined in our previously published literature [32].

The UPLC-QTOF/MS raw data (.wif) files were imported into Progenesis QI software (Waters Corporation, Milford, USA) for comprehensive data preprocessing, involving steps such as comparative review, peak selection, peak detection, and normalization. Concurrently, metabolite annotation was performed using the Human Metabolome Database (HMDB) and Kyoto Encyclopedia of Genes and Genomes (KEGG). The results generate a data matrix with retention times, mass-to-charge ratios, peak areas, etc.

The data matrix was further subjected to multivariate statistical analysis using SIMCA 14.1 software (MKS Data Analytics Solutions, Umea, Sweden), involving Principal Component Analysis (PCA) and latent structure discriminant analysis (OPLS-DA). To ensure model validity, the OPLS-DA model underwent testing with 200 permutations to assess for potential overfitting. Subsequently, metabolites with a *p*-value < 0.05 from the Student’s *t*-test and a VIP value > 1.0 from the OPLS-DA model were identified as differentially altered metabolites (DMs).

Metabolites were imported into MetaboAnalyst 5.0 for metabolic pathway enrichment, while pathway analysis was performed with reference to the KEGG database.

### 2.6. Transcriptomics Analysis

Total RNA extraction from gill tissue was carried out using Trizol reagent (Invitrogen, Carlsbad, CA, USA) following the manufacturer’s instructions. The quality of RNA samples was assessed for purity and concentration using a NanoDrop 2000 spectrophotometer. Additionally, the integrity of RNA was evaluated through agarose gel (1%) electrophoresis and an Agilent 2100 Bioanalyzer with the RNA Nano 6000 Analysis Kit (Agilent Technologies, CA, USA).

RNA-seq library preparation was performed using the TruSeq RNA kit. Subsequently, library sequencing was performed on the Illumina HiSeq 4000 platform. After sequencing, initial raw reads were preliminarily filtered. Mapped reads were aligned and assembled using Hisat2 and StringTie. Differential expression genes (DEGs) were identified by DESeq2 R packages. Genes with adjusted *p*-values < 0.05 and |log2 FC| ≥ 1 were considered DEGs.

Finally, Goatools and KOBAS were used for further enrichment analysis of DEGs, employing the Kyoto Encyclopedia of Genes and Genomes (KEGG) pathway and Gene Ontology (GO) databases.

### 2.7. Integrative Analysis of Metabolomics and Transcriptomics

Integrated analyses between treatment groups with distinct base concentrations involved both differentially altered metabolites (DMs; *p*-values < 0.05, VIP > 1) from metabolomics and differentially expressed genes (DEGs; *p*-adjust < 0.05, |log2 FC| ≥ 1) from transcriptomics. Pearson’s method was employed to calculate data correlation coefficients between metabolomics and transcriptomics using the R package. Heatmaps were generated to visually represent the association between DMs and DEGs.

### 2.8. Statistical Analysis

Statistical analysis of biochemical data was conducted using GraphPad Prism 9.0 (GraphPad Software Inc., San Diego, CA, USA). The Student’s *t*-test was applied to determine differences between the means of the data. Results were presented as means ± standard deviation (S.D.), and asterisks (*) denoted statistically significant differences between the experimental group and the control group, with * *p* < 0.05, ** *p* < 0.01, *** *p* < 0.001, and **** *p* < 0.0001.

## 3. Results

### 3.1. Observation of Gill Tissue of Triploid and Diploid Crucian Carp Under NaHCO_3_ Stress

**Figure 1:**
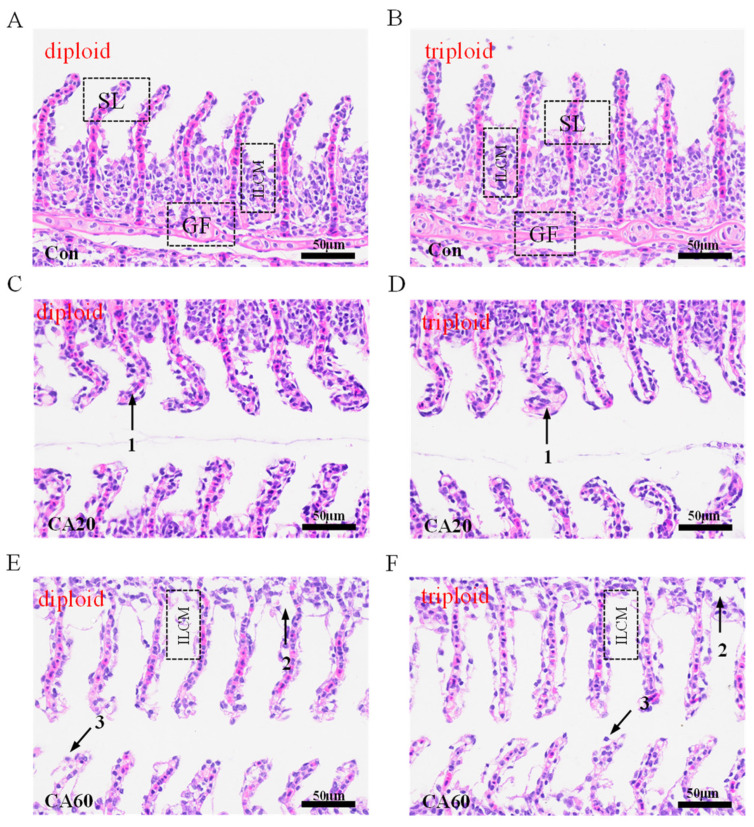
Effect of different concentrations of carbonate exposure on gill histology structure of diploid and triploid crucian carp. ILCM interlayer cells mass; GF. gill filament; SL. secondary lamella; 1. secondary lamella curved at the end; 2. Cellular vacuolation; 3. Epithelial rupture with hemorrhage: (**A**,**B**) Normal gill histology in diploid and triploid freshwater controls. (**C**,**D**) Diploid and triploid crucian carp gill 20 mmol/L NaHCO_3_ exposure groups. (**E**,**F**) Diploid and triploid crucian carp gill 60 mmol/L NaHCO_3_ exposure groups. (H&E, 400×, scale bar: 50 µm).

The impact of NaHCO_3_ stress on the histological structure of crucian carp gills is illustrated in Figure 1. In the control group, the gill filaments and secondary lamellae of crucian carp exhibited a structurally intact appearance. The gill filaments formed rod-like structures, with a cocked end, while the secondary lamellae extended perpendicularly to the gill filaments, resembling willow branches (Figure 1A,B). In the CA20 group, there was a noticeable curvature at the end of the secondary lamellae (Figure 1C,D). In the CA60 group, a significant reduction in the interlamellar cell mass, severe vacuolization of gill cells, shedding of necrotic epithelial cells in the secondary lamellae, and an overall deviation from the normal morphology of gill filaments were observed (Figure 1E,F).

### 3.2. Changes in Biochemical Parameters of Triploid and Diploid Crucian Carp Under NaHCO_3_ Stress

**Figure 2:**
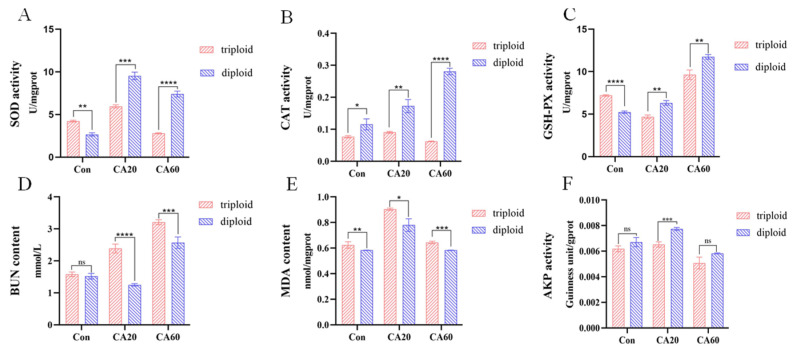
Changes in biochemical indicators in the gills of triploid and diploid crucian carp exposed to CA: (**A**) Gill SOD activity. (**B**) Gill CAT activity. (**C**) Gill GSH-PX activity. (**D**) BUN contents. (**E**) MDA contents. (**F**) Gill AKP activity. * *p* < 0.05, ** *p* < 0.01, *** *p* < 0.001, **** *p* < 0.0001. “ns” means that there is no significant difference between the two groups.

Figure 2 depicts the variations in biochemical parameters of triploid and diploid crucian carp under carbonate alkaline (CA) exposure. In the Con group, the superoxide dismutase (SOD) activity of triploids was significantly higher than that of diploids (*p* < 0.01). With increasing alkalinity, SOD activity initially increased and then decreased, remaining higher than that of the Con group. Notably, diploid SOD activity was significantly higher than that of triploids in the CA20 and CA60 groups (*p* < 0.001) (Figure 2A). Catalase (CAT) activity was consistently lower in triploids than in diploids at all three levels of alkaline concentration (*p* < 0.05). CAT activity in diploids increased with elevated alkaline concentration, while in triploids, it increased and then decreased, ultimately falling below control levels (Figure 2B).

In the Con group, glutathione peroxidase (GSH-Px) activity was significantly higher in triploids than in diploids (*p* < 0.0001). With increasing base concentration, GSH-Px activity in triploids exhibited a decrease followed by an increase, whereas GSH-Px activity in diploids continued to increase. In both the CA20 and CA60 groups, GSH-Px activity in diploids was significantly higher than in triploids (*p* < 0.01) (Figure 2C). Blood urea nitrogen (BUN) content did not significantly differ between triploid and diploid crucian carp in the Con group. However, BUN content increased with escalating alkalinity in the triploid group, reaching a peak in the CA60 group and decreasing before increasing again. In the diploid group, BUN content increased, ultimately being significantly higher than in the control group. Diploid BUN content was significantly lower than triploid in both CA20 and CA60 groups (*p* < 0.001) (Figure 2D).

Malondialdehyde (MDA) content in diploids was significantly lower than in triploids at all three levels of alkaline concentration (*p* < 0.05). It exhibited an increasing and then decreasing trend with rising alkaline concentration in both triploid and diploid crucians, eventually returning to control levels (Figure 2E). Alkaline phosphatase (AKP) activity in triploid and diploid crucian carp did not significantly differ in the Con group. In the CA20 group, AKP activity was significantly higher in diploids than in triploids (*p* < 0.01) before decreasing to control levels (Figure 2F).

### 3.3. Metabolomic Analysis of Triploid and Diploid Crucian Carp Under NaHCO_3_ Stress

Multivariate statistical analysis, including PCA and OPLS-DA, was employed to discern differences in gill metabolites of triploid and diploid crucian carp at various carbonate alkaline (CA) concentrations. The PCA score plots for both positive and negative ion modes are presented in Figure S1A,B. In the unsupervised principal component analysis model, metabolites were not distinctly differentiated between the experimental and control groups, potentially owing to limitations in the PCA model [34]. Notably, the QC samples exhibited robust clustering in smaller areas, indicating the high stability and reproducibility of the instrument. In the OPLS-DA scoring plot, a clear separation was observed between triploids and diploids at different CA concentrations, signifying significant differences in gill metabolism between triploid and diploid crucian carp under varying CA concentrations (Figure S1C–H).

The cumulative R2Y values of the OPLS-DA score plots for Con 2n vs. Con 3n, CA20 2n vs. CA20 3n, and CA60 2n vs. CA60 3n in the positive ion mode were 0.987, 0.97, and 0.992, respectively, with corresponding Q2 values of 0.939, 0.908, and 0.96. In the negative ion mode, the cumulative R2Y values were 0.953, 0.968, and 0.97, with Q2 values of 0.882, 0.881, and 0.847, respectively. These results indicate that the OPLS-DA model exhibited reliable fit and predictive power, rendering it suitable for subsequent data analysis. The OPLS-DA model underwent 200 permutations to ascertain whether it was overfitted (Figure S2). The results revealed that the random permutations produced higher R2 and Q2 values than the original OPLS-DA model, suggesting the model’s reliability and validity and confirming that it was not overfitted. Differential metabolites (DMs) were identified using criteria of VIP > 1 and *p*-value < 0.05. In Con 2n vs. Con 3n, 19 DMs (12 up-regulated and 7 down-regulated) were detected. For CA20 2n vs. CA20 3n, 28 DMs (9 up-regulated and 19 down-regulated) were identified. In CA60 2n vs. CA60 3n, 22 DMs (10 up-regulated and 12 down-regulated) were found. Table S1 provides detailed information about these DMs.

**Figure 3:**
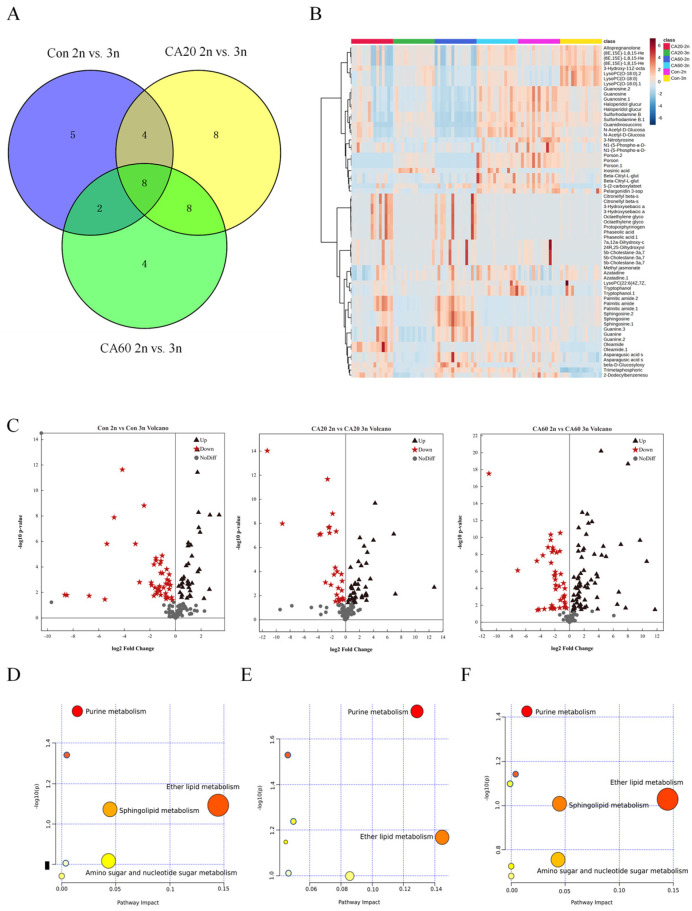
Differences in the metabolic profiles of triploid and diploid crucian carp after exposure to CA: (**A**) Venn diagram shows the overlap of DMs in triploid and diploid crucian carp in different alkalinities. (**B**) Hierarchical clustering based on the differential metabolites with *p* < 0.05 and VIP > 1.0. (**C**) The volcano plots show the distribution of DMs in the three CA treatment groups. (**D**–**F**) Significantly changed pathways based on the enrichment and topology analyses. Bubble size is proportional to the impact of each pathway, and bubble color denotes the degree of significance, from the highest (red) to the lowest (white).

The Venn diagram in Figure 3A illustrates the overlap of differential metabolites (DMs) in triploid and diploid crucian carp under different alkalinity conditions. The hierarchical cluster analysis in Figure 3B represents the content and relative relationships of the DMs, showing a significant change in DMs across various treatment groups. Volcano plots in Figure 3C depict the distribution of DMs in triploid and diploid crucian carp at different base concentrations. The results indicate that in the Con group, DMs were primarily enriched in metabolic pathways such as purine metabolism, ether lipid metabolism, sphingolipid metabolism, and amino and nucleotide sugar metabolism (Figure 3D). In the CA20 group, DMs were mainly enriched in purine metabolism, ether lipid metabolism, and sphingolipid metabolism (Figure 3E). In the CA60 group, DMs were primarily enriched in metabolic pathways such as purine metabolism, ether lipid metabolism, sphingolipid metabolism, and amino and nucleotide sugar metabolism (Figure 3F).

### 3.4. Transcriptomic Analysis of Triploid and Diploid Crucian Carp Under NaHCO_3_ Stress

**Figure 4:**
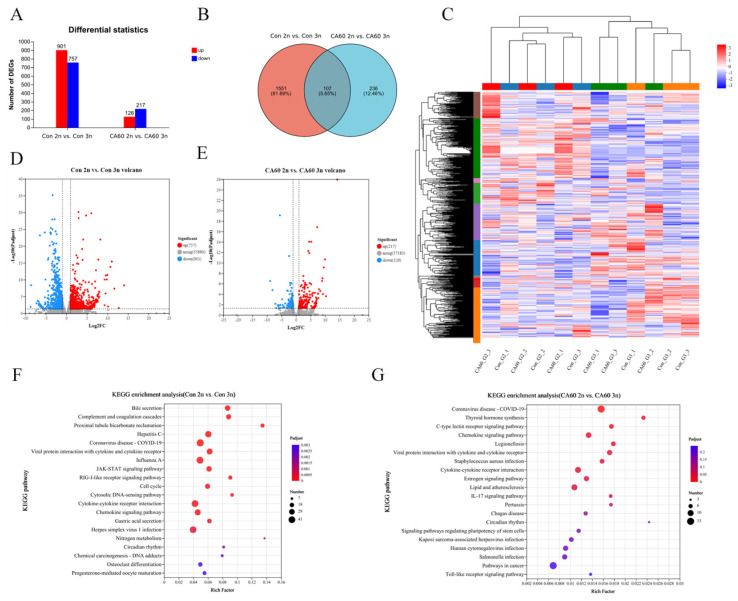
Transcriptome analysis of triploid and diploid crucian carp gills after CA stress: (**A**) The numbers of DEGs in gills after exposure to CA. (**B**) Venn diagram of DEGs between two CA-treated groups. (**C**) Hierarchical clustering analysis of DEG in two base concentration groups. (**D**) Volcano map of DEGs in the Con group. (**E**) Volcano plot of DEGs in the CA60 group. (**F**) KEGG enrichment analysis in the Con group. (**G**) KEGG enrichment analysis in the CA60 group.

Transcriptomic analyses were conducted on triploids and diploids in both the Con and CA60 groups to explore differences in gene transcription in triploid crucian carp under varying levels of alkaline stress. Table S2 provides details on the quality of the transcriptomics data. A total of 1658 differentially expressed genes (DEGs) were identified in the comparison between Con 2n vs. Con 3n (*p*-adjust < 0.05 and |log2 FC| ≥ 1). Out of these, 901 genes were up-regulated, and 757 genes were down-regulated in diploids compared to triploids. In the CA60 2n vs. CA60 3n comparison, 343 DEGs were identified, with 126 up-regulated and 217 down-regulated genes in diploids compared to triploids (Figure 4A). The expression levels of DEGs are detailed in Table S3. Volcano plots generated from the DEGs provide an overview of their distribution (Figure 4D,E). Additionally, a hierarchical cluster analysis was performed on the DEGs, visualized as a heat map, illustrating the expression differences between the four groups (Figure 4C). These results demonstrate significant differences in gene expression between triploid and diploid crucian carp in both the control and alkaline stress groups.

To further elucidate the association between differentially expressed genes (DEGs) and metabolic pathways, we conducted KEGG and GO enrichment analyses on all DEGs. In the KEGG enrichment analysis, several metabolic pathways were significantly enriched in both the Con 2n vs. Con 3n and CA60 2n vs. CA60 3n groups. Notable pathways included the C-type lectin receptor signaling pathway, IL-17 signaling pathway, Toll-like receptor signaling pathway, Nitrogen metabolism, and Complement and coagulation cascades (Figure 4F–G). Furthermore, the GO enrichment analysis for the CA60 2n vs. CA60 3n group revealed that the differential genes were categorized into three major branches of GO: biological processes, cellular components, and molecular functions. The analysis (Figure S3) indicated that the biological process was predominantly associated with chemokine activity and chemokine receptor binding. The main cellular components included collagen trimer and extracellular space, while histone H3-K4 demethylation, trimethyl-H3–K4-specific, and lipoprotein metabolic processes were prominent molecular functions. These findings suggest that triploid crucian carp exhibit significant differences in cellular metabolism and immune processes in response to CA60 stress.

### 3.5. Integrated Analysis of Metabolomics and Transcriptomics

**Figure 5:**
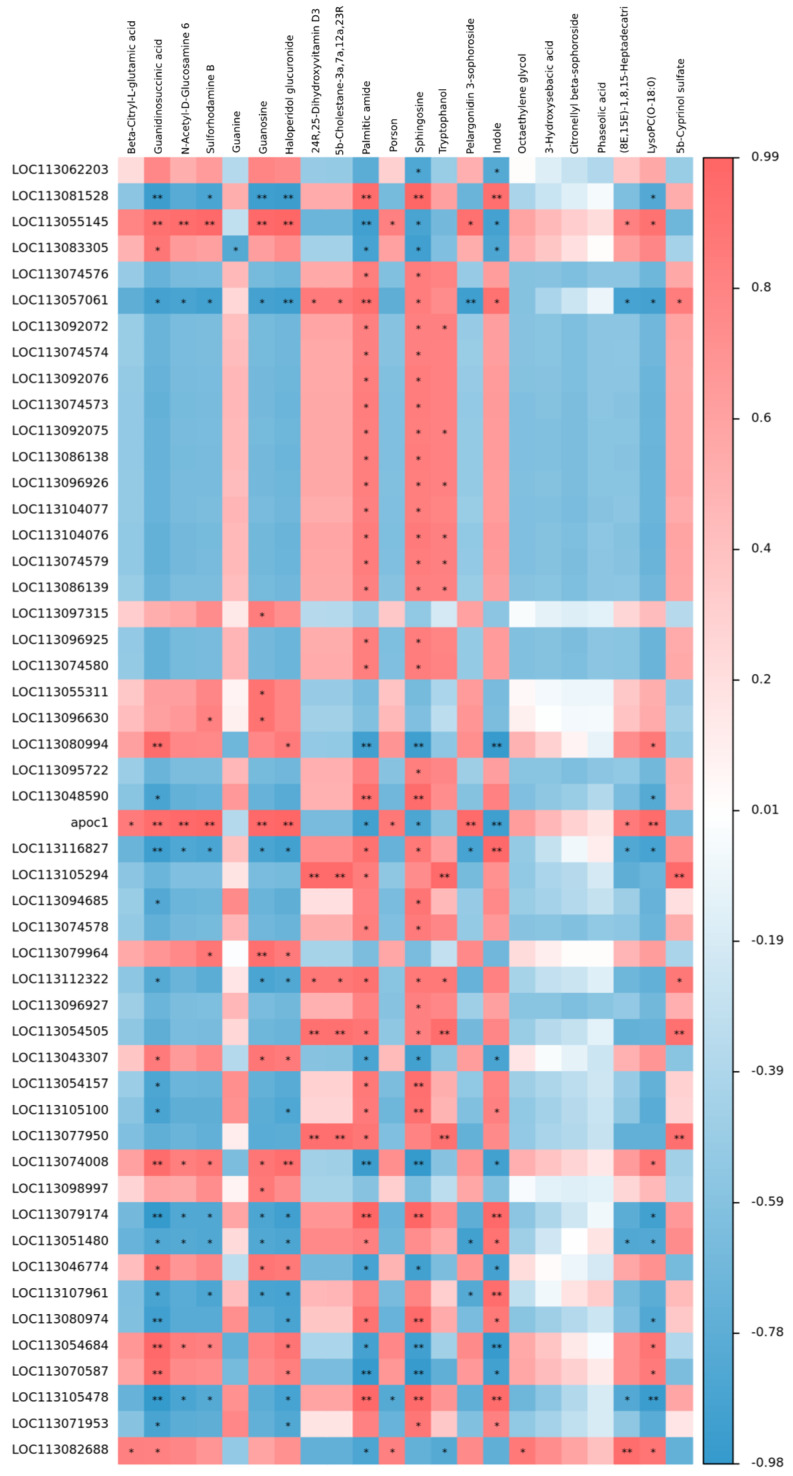
Heatmap of correlation between metabolomics and transcriptomics after CA60 stress. Heat maps of metabolites in columns and genes in rows show the relationship between genes and metabolites. Red and blue indicate positively correlated and negatively correlated related to transcriptomics and metabolomics. “*” indicates a significant difference, *p*-value < 0.05; “**” indicates a highly significant difference, *p*-value < 0.01.

**Figure 6:**
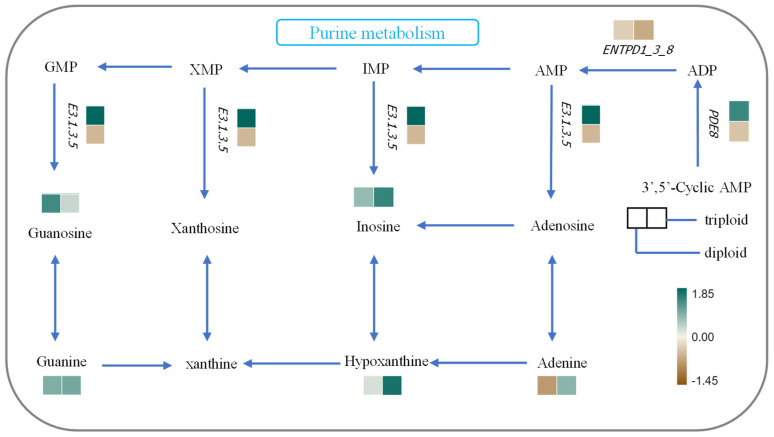
Changes in metabolites and expressed genes associated with purine metabolism in the gills of triploid and diploid crucian carp following exposure to CA. Genes are indicated in italics to distinguish them from metabolites.

The correlation between transcriptomics and metabolomics was revealed by using the Pearson method. An overview of the differentially expressed genes (DEGs) and differential metabolites (DMs) found between CA60 group triploids and diploids is depicted in Figure 5. This visualization demonstrates strong correlations between each transcript and metabolite. For instance, the DEG with the symbol LOC113083305 exhibited a robust negative correlation with guanine, a metabolite associated with the purine-rich metabolic pathway. Similarly, the DEG with the symbol LOC113048590 showed a strong positive correlation with sphingomyelin, a metabolite linked to the sphingolipid-rich metabolic pathway. These correlations underscore the specific associations between individual transcripts and metabolites (Figure 5). Figure 6 provides an overview of the relationship between DMs and DEGs in the purine metabolic pathway. This depiction further supports the notion that carbonate alkaline stress influences the gene–metabolite network in the gills of crucian carp.

## 4. Discussion

Alkaline water, characterized by its widespread distribution, high pH, carbonate alkalinity, and unbalanced proportions of major ions, poses a significant threat to the growth and development of aquatic organisms [35]. Diploid and triploid crucian carp differ biologically, resulting in markedly different environmental sensitivities [36]. Investigating distinct response mechanisms to alkaline stress is of paramount importance for the breeding of crucian carp varieties. In this study, we employed a comprehensive approach including histological examinations, biochemical analyses, and multi-omics methods to explore the different physiological and metabolic responses of diploid and triploid crucian carp to different concentrations of NaHCO_3_ stress. We aim to improve the understanding of the diverse regulatory mechanisms employed by diploid and triploid crucian carp in response to alkaline stress.

### 4.1. Effect of NaHCO_3_ Alkalinity on the Histological Structure of Triploid and Diploid Crucian Carp Gills

The impact of alkali stress on gill tissues has been documented in various studies, revealing significant structural changes. For instance, research on spotted sea bass (*Lateolabrax maculatus*) demonstrated a reduction in interlamellar cell mass and vacuolization of lamellar and filamentous epithelial cells after 72 h of exposure to 18 mmol/L NaHCO_3_, indicating severe damage to sea bass gill tissue [28]. Similarly, in grass carp (*Ctenopharyngodon idella*), prolonged exposure to NaHCO_3_ for 60 days resulted in a reduction in interlamellar cell mass, necrosis, and gradual detachment of the epithelium of the gill lamellae [37].

This research is consistent with these findings, showing a significant reduction in interlamellar cell mass, curvature at the end of gill lamellae, and the appearance of severe cell vacuolization in the CA60 group as alkalinity increased. This consistency of results suggests that oxidative damage has occurred in gill tissues, leading to impaired cell function. Oxidative stress is further indicated by the observed increase in antioxidant enzyme activity and elevated MDA levels. As alkalinity increased, the severity of oxidative damage deepened, resulting in irreversible injuries such as apoptosis, epithelial rupture, and detachment of gill tissue. This suggests that NaHCO_3_ stress has the potential to compromise the structural integrity of gill tissue, impairing respiratory function, and ultimately leading to hypoxia or even mortality.

### 4.2. Effect of NaHCO_3_ Alkalinity on Nitrogen Excretion in Triploid and Diploid Crucian Carp

Blood Urea Nitrogen (BUN) is a crucial metabolite associated with protein metabolism and amino acid balance in animals. In serum, BUN serves as an important indicator reflecting the state of protein metabolism. A lower BUN level is indicative of a well-balanced amino acid profile in fish, facilitating efficient synthesis of body proteins [38]. This research findings indicate that alkaline stress resulted in significantly elevated BUN levels in the blood of crucian carp compared to the control group. This suggests that alkaline stress led to an impairment of nitrogen excretion in crucian carp, resulting in an accumulation of BUN in the fish. These observations are in line with previous studies, such as in stingrays (*Himantura signifer*), where exposure to salinity stress led to a significant increase in the urea content of muscle tissue, indicating increased nitrogen retention and decreased urea nitrogen excretion capacity [39]. Interestingly, in the CA20 group, BUN levels were significantly lower in diploid carp compared to triploids. This finding implies that diploid crucian carp may possess a more efficient nitrogen excretion mechanism compared to triploids under the conditions of alkaline stress. The differences in BUN levels between diploid and triploid crucian carp highlight potential variations in their physiological responses to alkaline stress and underscore the importance of considering ploidy effects in such studies.

Purine metabolism is a vital component of cellular physiology with significant implications for the development of metabolic syndromes [40]. In aquatic animals, purine metabolism serves as a crucial pathway for the excretion of nitrogenous wastes. The process involves the conversion of guanine and adenine to xanthine, which is further metabolized to NH_3_ and CO_2_ through intermediates such as uric acid, allantoin, allantoic acid, and urea. These end products are then excreted from the body [41]. Our research findings reveal that purine metabolism was notably enriched in the gills of crucian carp under alkaline stress. Simultaneously, genes related to nitrogen metabolism exhibited significant changes in the gills. This is consistent with similar observations in *Scophthalmus maximus*, where purine metabolism was enriched, and related genes were up-regulated under phosphate stress, indicating a role in stress resistance and energy storage [42]. Moreover, in a study on Nile tilapia, dietary supplementation with LBP (*Lycium barbarum* polysaccharides) activated purine metabolism, leading to increased excretion of nitrogenous wastes and promoting growth [43]. The disruption of purine metabolism observed in crucian carp under alkaline stress suggests a link to nitrogen metabolism and a subsequent impact on protein synthesis functions. These findings contribute to a deeper understanding of the metabolic responses of crucian carp to environmental stressors, emphasizing the significance of purine metabolism in maintaining physiological balance under adverse conditions.

Our research elucidates the effects of NaHCO_3_ stress on purine metabolism in crucian carp, revealing the complex interplay between environmental stress, energy metabolism, and genetic responses. Purines, particularly guanine, play pivotal roles in various biological processes, such as nucleic acid synthesis and cellular energy metabolism [44,45]. Previous studies, including research on *Takifugu obscurus* gills under hypoxic stress, have indicated that hypoxic conditions can activate detoxification and nitrogen excretion genes. Additionally, metabolic pathways such as purine metabolism are activated to meet the energy requirements for nitrogen assimilation and utilization [46]. Our study reveals that NaHCO_3_ stress disrupts purine metabolism in crucian carp gills, with downstream differential metabolites (Inosine, Hypoxanthine, Guanosine, Guanine, and Adenine) and differentially expressed genes (*ENTPD1_3_8*, *PDE8*, and *E3.1.3.5*) associated with purine metabolism showing significant differences between diploid and triploid crucian carp. The down-regulation of genes related to purine metabolism in diploids, compared to triploids, suggests that diploids may experience less damage from environmental stress. The excess energy in diploids could be channeled toward DNA replication, RNA production, and protein synthesis during cell proliferation, promoting protein metabolism and amino acid balance. This may explain the significantly lower BUN levels in diploids compared to triploids. This paper underscores the intricate connections between environmental stress, metabolic pathways, and genetic responses in crucian carp, providing valuable insights into the adaptive mechanisms of different ploidy crucian carp under NaHCO_3_ stress.

### 4.3. Effect of NaHCO_3_ Alkalinity on Lipid Metabolism in Triploid and Diploid Crucian Carp

Under physiological conditions, there is a delicate balance between the production of reactive oxygen species (ROS) and the antioxidant defense system. Elevated levels of ROS, when accumulated in cells, prompt rapid interactions between polyunsaturated fatty acids on the cell membrane and free radicals, initiating the process of lipid peroxidation and producing by-products such as malondialdehyde (MDA) [47]. MDA is a key measure of the extent of peroxidative damage to tissue [48]. Our investigation revealed a significant elevation in MDA content in both triploid and diploid crucian carp exposed to CA20 concentration, with a subsequent declining trend observed with increasing alkalinity. In all three groups, diploid MDA levels were consistently lower than triploids. In contrast, Han et al. [49] documented a notable surge in MDA levels in both triploid and diploid rainbow trout under moderate ammonia stress, whereas MDA levels exhibited a diminishing trend under high ammonia stress, with diploid rainbow trout displaying higher MDA levels than triploid counterparts. These findings suggest diverse stress responses among fish species facing distinct environmental stressors. In summary, our study indicates that alkaline stress compromises the antioxidant defense mechanisms in crucian carp, disrupts physiological homeostasis, induces lipid peroxidation damage, and underlines the superior antioxidant capacity of diploid crucian carp compared to triploids.

Disorders of lipid metabolism, which are influenced by intricate interactions between genetic, behavioral, and environmental factors, represent a global challenge to public health [50]. Transcriptomic studies in *Nile tilapia* and *Leuciscus waleckii* under alkaline stress revealed the responsiveness and adaptability of genes associated with lipid metabolism and detoxification [21,51]. In our investigation, sphingolipid metabolism emerged as a key pathway, with significant enrichment of differential metabolites in diploid crucian carp compared to triploids, accompanied by a noteworthy down-regulation of sphingomyelin content. Transcriptomics data further indicated significant down-regulation of genes linked to sphingolipid metabolism, including *GNA13* and *FCER1G*, in diploid crucian carp. Sphingolipids, amphiphilic lipids with a sphingosine backbone, form lipid rafts that regulate membrane transport and mediate signaling [52]. Sphingomyelin, a fundamental component, can contribute to ceramide formation, inducing apoptosis [53]. Alkaline stress-induced disturbances in sphingolipid metabolism, observed in crucian carp gills, were associated with apoptosis and posed a significant threat to survival [29]. *GNA13* involved in osteolysis and skeletal development may have an effect on individual growth [54], while *FCER1G*, an innate immunity gene, plays a role in various diseases [55]. The down-regulation of *GNA13* and *FCER1G* expression in diploid crucian carp, compared to triploids, suggests a reduced inflammatory response and immune activity. Simultaneously, the reduction in sphingolipid content in diploid crucian carp may serve as a preventive measure against excessive apoptosis, contributing to the maintenance of normal physiological functions in gill cell membranes.

### 4.4. Effect of NaHCO_3_ Alkalinity on the Antioxidant Capacity of Triploid and Diploid Crucian Carp

The production of reactive oxygen species (ROS) and the antioxidant defense system are in a delicate equilibrium balance under normal circumstances [56]. Environmental variables such as salinity, temperature, oxygen levels, ammonia, nitrite, and pH changes can disrupt this balance, leading to an excessive generation or accumulation of ROS, resulting in oxidative stress [57]. Crucial antioxidant enzymes, such as superoxide dismutase (SOD) and catalase (CAT), form the SOD-CAT system, which serves as the primary defense against ROS formation during stress and plays a pivotal role in neutralizing ROS to protect cells from oxidative stress [58]. In conditions of substantial ROS accumulation, SOD converts excess superoxide ions (O_2_^−^), and hydrogen peroxide (H_2_O_2_) is subsequently reduced to oxygen (O_2_) and water (H_2_O) by CAT, effectively counteracting the effects of oxidative stress [59]. Glutathione peroxidase (GSH-Px) helps to prevent the production of ROS by neutralizing. Our study revealed that both species of crucian carp exhibited an increasing and then decreasing trend in SOD activity, with diploid crucian carp displaying significantly higher SOD activity than triploid carp in the alkaline concentration group. CAT activity in diploids increased continuously, significantly exceeding triploids. GSH-Px activity in diploids demonstrated a decreasing then increasing pattern, consistently higher than in triploids. These findings are consistent with studies by Shi et al. [60], where SOD, CAT, and GSH-Px activities exhibited similar trends in rainbow trout under acute high-temperature stress, indicating intense oxidative stress and a robust antioxidant response. Similarly, Han et al. [49] reported elevated SOD activity in triploid rainbow trout under different concentrations, with CAT activity showing a comparable increasing-then-decreasing pattern in the low-concentration group.

We propose that the surge in SOD and CAT activities results from external environmental stress, triggering the activation of the antioxidant system in crucian carp. As the alkaline concentration increases, the excessive accumulation of ROS or H_2_O_2_ overwhelms the fish’s endogenous antioxidant defense system, ultimately leading to the inactivation or destruction of SOD and CAT. Significantly higher SOD, CAT, and GSH-Px activities in diploids compared to triploids indicate a superior antioxidant capacity in diploids, allowing them to respond more rapidly to environmental stressors.

### 4.5. Effect of NaHCO_3_ Alkalinity on the Immunity of Triploid and Diploid Crucian Carp

AKP, essential for maintaining calcium and phosphorus homeostasis and regulating lipid metabolism in fish, plays a pivotal role in both immune and metabolic functions [61]. In response to acute salt stress, AKP activity in *Litopenaeus vannamei* exhibited fluctuations, notably increasing and subsequently decreasing significantly within 24 h following a sudden decrease in water salinity [62]. A study on *Larimichthys crocea* demonstrated a significant decline in AKP activity in the spleen two weeks after various salinity treatments, indicating a negative impact on immune function due to chronic stress [63]. In our study, gill AKP activity initially increased in both diploid and triploid crucian carp and then decreased significantly to below control levels. Moreover, diploid carp displayed significantly higher AKP activity compared to triploids, suggesting enhanced immunity. In the CA20 group, diploids exhibited significantly elevated AKP activity compared to triploids, emphasizing their superior immunity. Conversely, in the CA60 group, AKP activity showed no significant difference between the two crucian carp species, possibly due to the high alkali concentration and the negligible effect on immunity caused by ploidy effects.

*IL-17*, a vital cytokine produced by activated memory T cells, plays a crucial role in inflammation and immune responses [64]. The IL-17 signalling cascade response begins with the binding of IL-17 cytokines to their receptors IL-17RA and IL-17RC [65]. This pathway exerts antiviral effects by activating the expression of pro-inflammatory and anti-microbial infection genes, such as *IL-1β*, *TNF-α*, and *IL-8* [66]. *IL-8*, known for attracting neutrophils to sites of inflammation, plays a pivotal role in mediating inflammatory and immune responses [67]. Previous studies in grass carp indicated a significant increase in *IL-17* protein expression over time, suggesting its involvement in the host’s defense against viral infections [68]. In our study, transcriptomic data revealed significant enrichment of the *IL-17* signaling pathway in diploids compared to triploids, accompanied by a noteworthy down-regulation of *IL-8*. Additionally, Fan et al. [9] observed a significant up-regulation of *IL-8* in the intestine of Songpu mirror carp with increasing carbonate levels. These findings suggest that alkaline stress activates the *IL-17* signalling pathway in carp, promoting the expression of immune factors to mount an immune response against oxidative stress. The lower expression of *IL-8* in diploids compared to triploids indicates a milder inflammatory response in diploids and underlines their superior immune capacity. Thus, changes in immune factors serve as crucial indicators to assess the differential immune capabilities of triploid crucian carp under carbonate alkalinity exposure.

Cytokines, small peptides, or glycoproteins synthesized and secreted by various tissue cells, primarily immune cells, play a crucial role in regulating cell growth, differentiation, and immune responses [69]. Toll-like receptor signalling pathways (TLRs) are integral to the innate immune response and rapidly activate innate immunity by inducing the production of pro-inflammatory cytokines [70]. In our investigation, immune-related Differentially Expressed Genes (DEGs) were significantly enriched in the Toll-like receptor signaling pathway and cytokine–cytokine receptor interactions in diploid crucian carp compared to triploid crucian carp. In particular, chemokines and pro-inflammatory factors (*CXCL8*, *CXCR4*, *IL6R*, *CCL19*) exhibited significant down-regulation in diploids. Previous studies, such as Zhou et al. [71] on Nile tilapia and Wang et al. [72] on flounder, have also reported significant enrichment of Toll-like receptor signaling pathways and cytokine–cytokine receptor interactions in response to infection. These findings are consistent with our results and suggest that crucian carp activate Toll-like receptor signaling pathways and cytokine–cytokine receptor interactions to mount an immune response against oxidative stress induced by carbonate alkalinity (CA) stress. The down-regulation of chemokines and pro-inflammatory factors in diploids indicates a milder inflammatory response, suggesting that diploids may not elicit a stronger immune response in the same stressful environment.

## 5. Conclusions

This study elucidates the different adaptive mechanisms of diploid and triploid crucian carp facing saline–alkaline habitats by applying untargeted metabolomics, transcriptomics combined with histological analysis, and biochemical detection. Under saline–alkaline stress, the crucian carp showed damage to the gill tissue. Diploid crucian carp activated purine metabolism to excrete excess ammonia and exhibited superior nitrogen excretion capacity. At the same time, diploid crucian carp mobilized more energy to resist saline–alkaline stress and for organism growth and development. Notably, diploid crucian carp demonstrated exceptional antioxidant capacity and inflammation regulation in response to oxidative stress caused by saline–alkaline stress compared to triploid crucian carp. The above results all indicate that diploid crucian carp have better adaptability to saline–alkaline waters. These findings provide constructive insights to analyze the physiological response mechanisms of different ploidy of crucian carp under saline–alkaline stress, and are of great significance for species selection of different ploidy of crucian carp for aquiculture in saline–alkaline waters.

## Figures and Tables

**Table 1:** Experimental design.

2n	3n
Con	Con
CA20	CA20
CA60	CA60

## Data Availability

The data presented in this study are available on request from the corresponding author. The data are not publicly available due to privacy.

## Supplementary Materials

The following supporting information can be downloaded at: www.mdpi.com/article/10.3390/metabo15010005/s1, Table S1: Summary statistics for metabolomics differential metabolites; Table S2: Shows the quality of the transcriptomics data; Table S3: Shows DEGs expression levels. Figure S1: The PCA and OPLS-DA score plots for the positive and negative ion modes; Figure S2: The OPLS-DA model was subjected to 200 permutations for determining whether it was overfitted; Figure S3: Shows GO enrichment analysis of differentially expressed genes in CA20 and CA60 treatment groups.
