# Supplementary material for: Exploring Disparities in Gill Physiological Responses to NaHCO3-Induced Habitat Stress in Triploid and Diploid Crucian Carp (Carassius auratus): A Comprehensive Investigation Through Multi-Omics and Biochemical Analyses
Source: Metabolites. 2024 Dec 30;15(1):5. doi: 10.3390/metabo15010005 (PMC11767977; doi:10.3390/metabo15010005)
Supplement: Supplementary file 1 [file metabolites-15-00005-s001.zip › Figure S1.pdf]

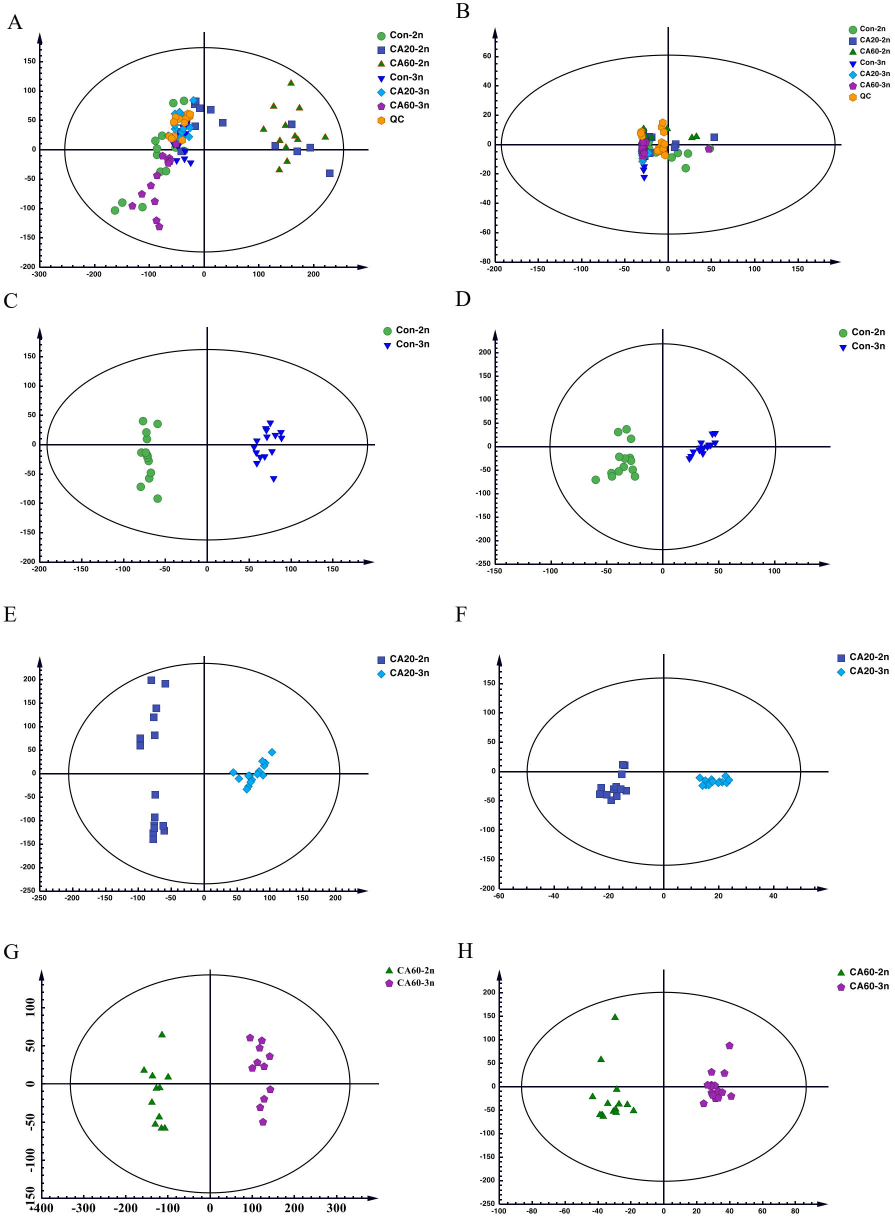

Figure S 1 PCA and OPLS-DA scores of diploid and triploid crucian carp gills under alkaline stress in positive (A,C, E, G) and negative (B,D, F, H) ion patterns
