# Supplementary material for: Exploring Disparities in Gill Physiological Responses to NaHCO3-Induced Habitat Stress in Triploid and Diploid Crucian Carp (Carassius auratus): A Comprehensive Investigation Through Multi-Omics and Biochemical Analyses
Source: Metabolites. 2024 Dec 30;15(1):5. doi: 10.3390/metabo15010005 (PMC11767977; doi:10.3390/metabo15010005)
Supplement: Supplementary file 1 [file metabolites-15-00005-s001.zip › Figure S2.pdf]

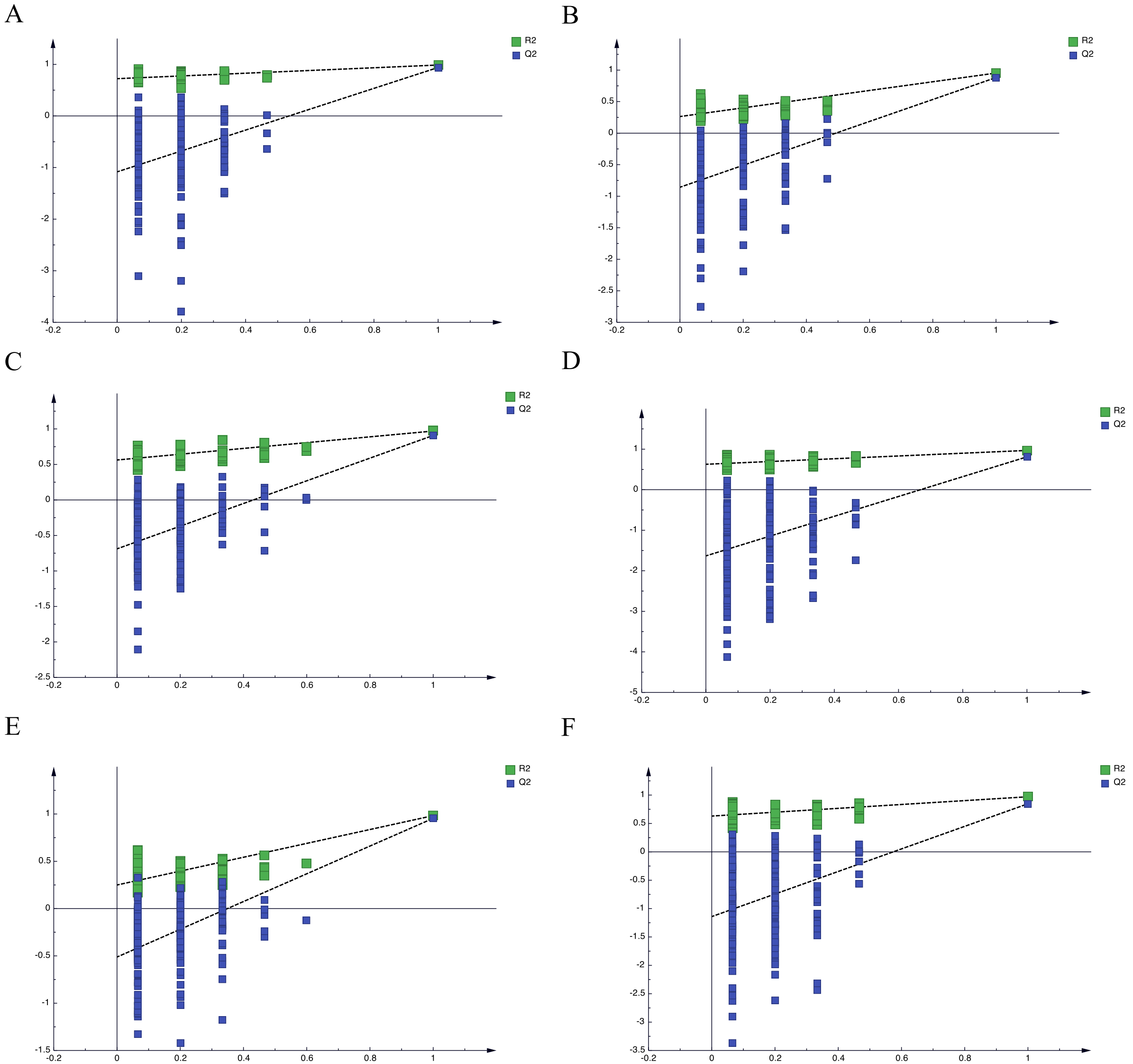

Figure S 2 OPLS-DA score plot and sequencing validation in positive and negative ion mode

(A&B) Con-2n and Con-3n groups in positive and negative ion mode ranking validation plots; (C&D) CA20-2n and CA20-3n groups in positive and negative ion mode ranking validation plots; (E&F) CA60-2n and CA60-3n groups in positive and negative ion mode ranking validation plots.
