# Supplementary material for: Exploring Disparities in Gill Physiological Responses to NaHCO3-Induced Habitat Stress in Triploid and Diploid Crucian Carp (Carassius auratus): A Comprehensive Investigation Through Multi-Omics and Biochemical Analyses
Source: Metabolites. 2024 Dec 30;15(1):5. doi: 10.3390/metabo15010005 (PMC11767977; doi:10.3390/metabo15010005)
Supplement: Supplementary file 1 [file metabolites-15-00005-s001.zip › Figure S3.pdf]

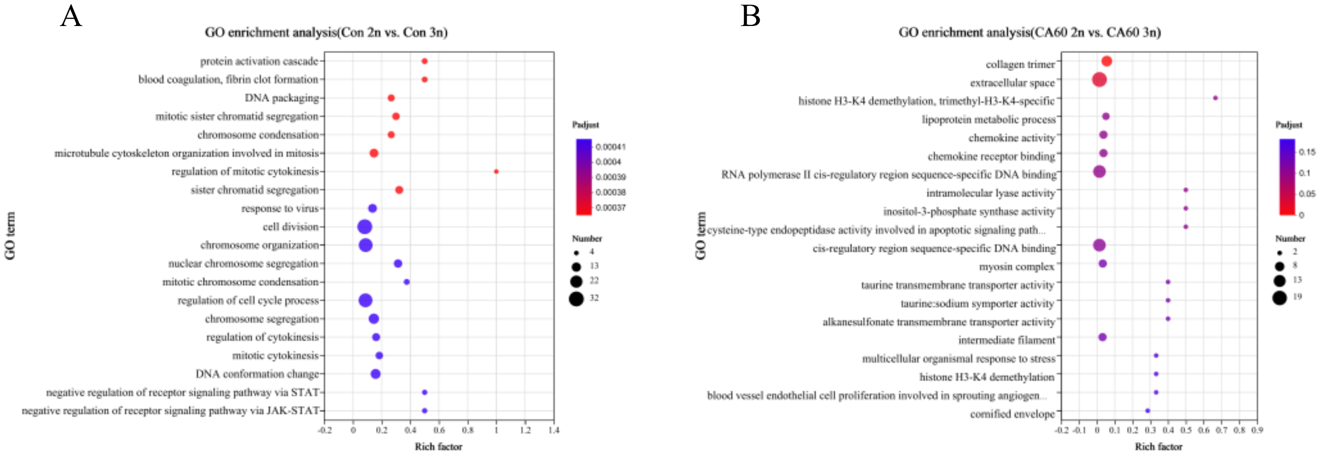

Figure S3 GO enrichment analysis of DEGs in the Con group (**A**) GO enrichment analysis of DEGs in the CA60 group （**B**）
